# Cytokine Production by Peripheral Blood CD4^+^ and CD8^+^ T Cells in Atopic Childhood Asthma

**DOI:** 10.1155/2010/606139

**Published:** 2010-12-08

**Authors:** Edyta Machura, Bogdan Mazur, Malgorzata Rusek-Zychma, Malgorzata Barć-Czarnecka

**Affiliations:** ^1^Department of Pediatrics, Medical University of Silesia, Ulica 3-go Maja 13-15, 41-800 Zabrze, Poland; ^2^Department of Microbiology and Immunology, Medical University of Silesia, Ulica Jordana 19, 41-808 Zabrze, Poland

## Abstract

There are conflicting studies on T cell cytokine production in childhood asthma. In this study intracellular cytokine expression of IL-2, IL-4, IL-10, IL-13, IFN-*γ*, and TNF-*α* in CD4^+^ and CD8^+^ T cells in children with atopic asthma were measured by flow cytometry. *Results*. A significant increase in the percentage of CD4^+^ and CD8^+^ T cells producing IL-4 and IL-13 and decrease in the percentage of CD4^+^ producing IFN-*γ* in asthmatic children was found. The percentage of CD4^+^/IL-13^+^ was significantly higher in severe asthma than in children with intermittent disease symptoms. Severity of asthma was associated with increased both serum IgE and frequencies of CD4^+^/IL-13^+^ T cells, as well as duration of disease. Moreover, a decrease in FEV_1_, FEV_1_/FVC was observed in relation to the severity of asthma. Changes in cytokine profile in CD8^+^ subpopulation didn't depend on the severity of the disease. *Conclusions*. Increased production of IL-4 and IL-13 in both CD4^+^ and CD8^+^ T cells accompanied by decreased IFN-*γ* expression in CD4^+^ T cells may be evidence that both lymphocyte subpopulations are implicated in the pathogenesis of asthma. Relationship of CD4^+^/IL-13^+^ T cells with disease activity suggests that this lymphocyte subset may have a prominent role in childhood asthma.

## 1. Introduction

Allergic asthma is one of the most common diseases in childhood which is caused by a combination of genetic and environmental factors [1]. A number of studies have shown the important role of activated memory CD4^+^ T cells as the main producer of Th2 cytokines in asthma and other atopic diseases [2, 3]. Th2 cytokines such as IL-4 and IL-13 interact with resident lung cells, including airway epithelium, myofibroblast, and smooth muscle cells, to induce the asthmatic phenotype [3]. These cytokines are the cause of pathophysiological features of asthma including airway inflammation, mucus secretion, and airway hyperresponsiveness. The production of Th2 cytokines was originally ascribed to CD4^+^ T cells, but a number of studies provided evidence that CD8^+^ T cells are able to secrete Th2 cytokines and are also essential for allergic inflammation and airway sensitivity [4, 5]. Although most of the studies on the activity of T cells cytokines in asthma revealed upregulated expression of Th2 cytokines at the site of allergic inflammation, as well as in circulating peripheral blood T cells, a recent study suggested that Th1 cells secreting IFN-*γ* might cause severe airway inflammation [4]. Regulatory T cells (Treg) may play a critical role in controlling the development of asthma, as they can suppress a potentially harmful immune response. There is evidence that the number and function of two major subsets of Treg, namely, CD4^+^CD25^+^Foxp3^+^ Tregs and IL-10 producing Tregs, are impaired or altered in patients with atopic asthma compared with healthy individuals [6].

Until now, only a few studies have directly identified different subsets of peripheral blood and airway T cells in children with asthma, and more specifically with respect to intracellular cytokines production, and the results are conflicting [7–10].

The aim of this study was to assess differences in cytokine profile in peripheral CD4^+^ and CD8^+^ T cells between children with asthma and healthy controls and to determine whether increasing severity of asthma is related to cytokine production.

## 2. Material and Methods

The study group comprised of 40 children (aged 5.2 to 15.8 years; mean age 9.2 ± 0.35 years) with allergic asthma, of whom 10 had intermittent, 14 mild, 12 moderate, and 4 had severe persistent asthma. The diagnosis of asthma and the assessment of severity were done according to the GINA 2002 criteria [11]. All children had a history of recurrent episodes of airway obstruction. Children above 6 years of age underwent spirometric assessment and presented reversibility of airway obstruction, as documented by positive response to a bronchodilator of at least 12% increase of forced expiratory volume in one second (FEV_1_). All children had positive skin prick tests (SPT) to one or more allergens (SPT was regarded as positive when mean diameter was at least 3 mm). The degree of allergic sensitization was measured by wheal size of skin prick tests. Thirty children with mild-to-severe persistent asthma were treated with regularly inhaled glucocorticoids (ICS), but with variable daily dose required to control the symptoms (at the time of evaluation, daily ICS dose ranged from 100 to 1000 *μ*g/day, mean daily dose: 311.0 ± 25.6). Duration of ICS treatment ranged from two months to 11 years (mean 4.5 ± 0.6). All children with asthma were well controlled; children with exacerbations were excluded from the study. Detailed data of asthma duration and ICS treatment were obtained from medical records and specific questionnaire. The control group consisted of 18 healthy children (aged 4.7 to 16.0 years; mean age 9.2 ± 0.7 years) with a negative history of allergic disease, normal level of total serum IgE, and negative results of skin prick test to a panel of aeroallergens (dust mite, mixed grass or tree pollen, cat, and dog; Allergopharma, Reinbek, Germany). Children included into the control group attended the outpatient pediatric clinic for nonimmunological, noninflammatory health problems and needed venous puncture.

The present study was approved by Ethics Committee of the Medical University of Silesia in Katowice and written informed consent was obtained from children's parents. Venous blood samples were obtained from each subject.

### 2.1. Total Serum IgE Level

Total serum IgE concentration was determined with the sandwich method which uses anti-IgE antibodies adsorbed on the microplates and a conjugate of alkaline phosphatase labeled goat anti-IgE antibody. The serum samples were incubated on microplates with anti-IgE antibody conjugate. The complexes IgE-anti-IgE were detected adding substrate p-nitrophenylphosphate (PNPP), and chromatic product was measured with photometry at 405 nm (ELISA kits, Allergopharma).

### 2.2. Intracellular Cytokine Staining

**Figure 1:**
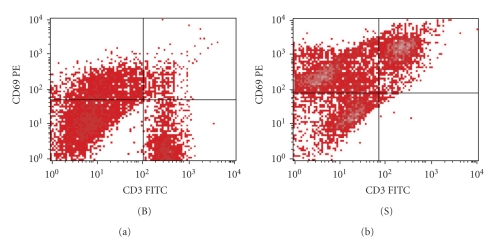
Flow cytometric expression of CD69 (activation marker) on CD3^+^ T cells before (B) and after polyclonal stimulation (S) of one patient with mild asthma (male, age 9.8 yrs).

Intracellular cytokine staining before and after polyclonal stimulation was assessed. Heparinized whole blood was diluted with RPMI (BioWhittaker) and 2% calf serum and after the addition of 25 *μ*l (25 ng/ml) of phorbol 12-myristate 13-acetate (PMA; Sigma), 20 *μ*l (1 *μ*g/ml) of ionomycin (Sigma), and 10 *μ*l (2.5 *μ*M) of monensin (Sigma) incubated in a water bath for 4 h at 37°C. Stimulated whole blood was distributed in 100 *μ*l aliquots into tubes containing 20 *μ*l antihuman CD3-FITC and CD4-PerCp (Becton Dickinson), vortexed, and incubated for 20 min at room temperature in the dark. To fix cells, 100 *μ*l of IntraPrep Reagent 1 (DAKO) was added to the samples followed by vortexing and incubation for 15 min at room temperature in the dark and washing with 2 ml of cell-wash solution. Simultaneous permeabilization of white cells and lysis of red cells was then carried out by adding 100 *μ*l IntraPrep Reagent 2 (DAKO) and incubation. Intracellular staining procedure included addition of 20 *μ*l of anti-IL-2, IL-4, IL-10, IL-13, IFN-*γ*, and TNF-*α* monoclonal antibodies or isotope control mouse antibodies conjugated with PE (Coulter Immunotech). The flow cytometric assessment with FACScan flow cytometry (Becton Dickinson) was performed on the same day. Acquisition was gated on CD3^+^CD4^+^ and CD3^+^CD8^+^ cells, and then the percentage of CD3^+^CD4^+^ and CD3^+^CD8^+^ cells producing IL-2, IL-4, IL-10, IL-13, IFN-*γ*, and TNF-*α* was determined on dot plots. Quadrants were set based on isotope control. The acquisition was performed both on samples stimulated with PMA and ionomycin, and on nonstimulated samples, in order to estimate the residual intracellular expression of the cytokines. Activation of the cells was confirmed by estimation of the CD69 expression, which was over 95% in all samples (Figure 1). Acquisition and analysis were performed with Cell Quest software package (Becton Dickinson).

### 2.3. Statistical Analyses

Statistical analyses were performed using software package (Statistica, version 3.0) and data presented as mean value ± SE. Comparisons between groups were made with the Kruskal-Wallis test, followed by Mann-Whitney U test. Correlations were calculated using the Spearman rank test. Values of *P* < .05 were considered as statistically significant.

## 3. Results

The demographics and background characteristics of the study groups are shown in Table 1. Both groups were similar with regard to age. 

### 3.1. Comparison of Eosinophil Counts and Serum IgE Levels between Children with Asthma and Control Group

The percentage as well as the count of peripheral blood eosinophils was significantly elevated in asthma children as compared with control group (*P* < .001). Total serum IgE levels were significantly higher in children with asthma than in controls (*P* < .01).

### 3.2. Frequencies of CD*4^+^* and CD*8^+^* T Cells Spontaneously Producing IL-2, IL-4, IL-10, IL-13, IFN-*γ*, and TNF-*α* in Children with Asthma and Control Group

Mean values of frequencies of CD4^+^ and CD8^+^ T cell spontaneously producing IL-2, IL-4, IL-10, IL-13, IFN-*γ*, and TNF-*α* are shown in Table 2. Percentage proportions of CD4^+^ T cell staining positively for IL-2, IL-4, IL-10, IL-13, IFN-*γ*, and TNF-*α* in unstimulated whole blood were similar in children with asthma compared to healthy controls. Frequencies of CD8^+^ T spontaneously producing IL-4 were significantly higher in children with asthma than in healthy controls (*P* < .05). No significant difference in remaining cytokine expression by unstimulated CD8^+^ T cells between asthma and control was observed. 

### 3.3. Frequencies of CD*4^+^* and CD*8^+^* T Cell Producing IL-2, IL-4, IL-10, IL-13, IFN-*γ*, and TNF-*α* after In Vitro Stimulationwith Phorbol 12-Myristate 13-Acetate and Ionomycin in Asthma Patients and Control Group

Mean values of frequencies of *CD4^+^* and *CD8^+^* T cell producing IL-2, IL-4, IL-10, IL-13, IFN-*γ*, and TNF-*α* after *in vitro *stimulation with phorbol 12-myristate 13-acetate and ionomycin are shown in Table 3. Frequencies of *CD4^+^* and *CD8^+^* T cells producing IL-4, IL-13 after *in vitro* stimulation were significantly higher in asthma patients than in the control group (IL-4: *P* < .00001, *P* < .00003 for *CD4^+^* and *CD8^+^* T cells, resp., IL-13: *P* < .00001 for *CD4^+^* and *CD8^+^* T cells). In contrast, frequencies of *CD4^+^* producing IFN-*γ* were significantly reduced in children with asthma compared to healthy subjects (*P* < .0001). Frequencies of *CD4^+^* staining positively for IL-2, IL-10, and TNF-*α*, and *CD8^+^* T cells staining positively for IL-2, IL-10, TNF-*α*, and IFN-*γ* after *in vitro* stimulation with phorbol 12-myristate 13-acetate and ionomycin were similar in both groups. 

**Figure 2:**
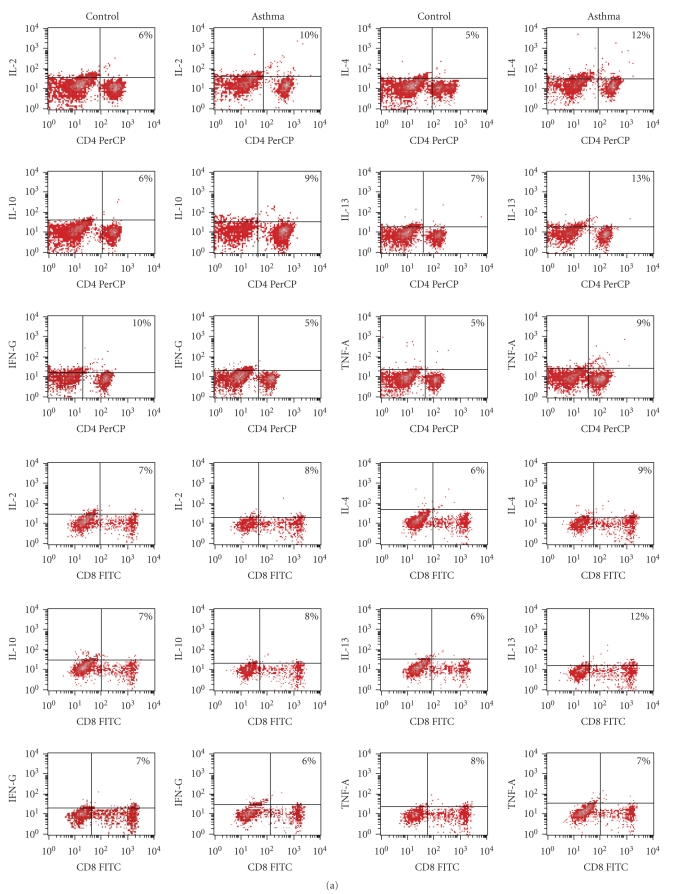

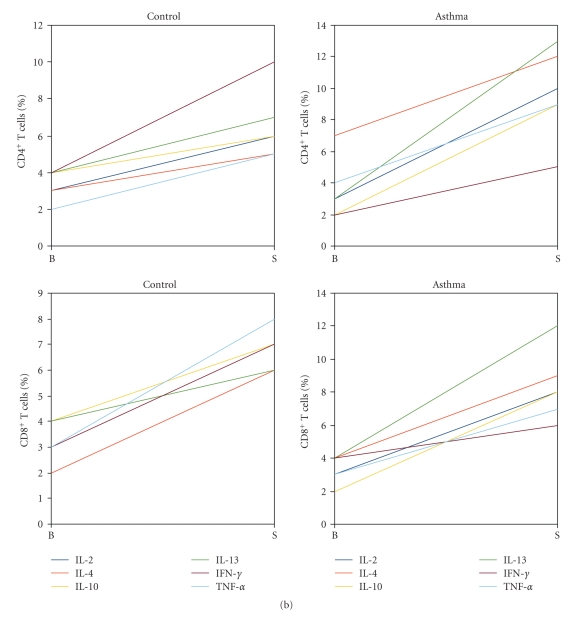
(a) Flow cytometry of the cytokine production values of IL-2, IL-4, IL-10, IL-13, INF-*γ*, and TNF-*α* in CD4^+^ and CD8^+^ T subpopulations. Example of one patient with mild asthma and one control. (b) Plots of CD4^+^ and CD8^+^ T cells producing various cytokines before and after polyclonal stimulation of one patient with mild asthma (male, age 9.8 yrs) and one control (male, age-9.5 yrs). B-baseline, spontaneous cytokine staining without stimulation (in the presence of monensin alone), S-cytokine staining with stimulation (unseparated whole blood from asthma patients and healthy controls was stimulated for 4 h with PMA and ionomycin, and production of IL-2, IL-4, IL-10, IL-13, INF-*γ*, and TNF-*α* of CD4^+^ and CD8 T^+^cells was determined at the single cell levels by intracellular cytokine staining and flow cytometry as described in Section 2).

An example of flow cytometry of the examined cytokine production values in CD4^+^ and CD8^+^ T subpopulations for patient with mild asthma and one control are shown in Figure 2.

### 3.4. Frequencies of *CD4^+^* and *CD8^+^* T Cell producing IL-2, IL-4, IL-10, IL-13, IFN-*γ*, and TNF-*α* before and after In Vitro Stimulation with Phorbol 12-Myristate 13-Acetate and Ionomycin in Patients with Different Degree of Severity of Asthma

Mean value of levels of cytokines in each group asthmatic children are shown in Table 4. After separating the patients according to the severity of disease (intermittent, mild persistent, moderate persistent, and severe persistent asthma) and then comparing the four groups, there was a significant increase in frequencies of *CD4^+^* T cells producing IL-13 following stimulation in children with severe asthma compared to those with intermittent asthma (*P* < .04). Also, in the group with severe asthma frequencies of polyclonaly stimulated *CD4^+^* T cells producing IL-10 tended to be higher compared to all three remaining subgroups, but the difference did not reach statistical significance (*P* > .07). Additionally, frequencies of *CD4^+^* T cells spontaneously producing IL-10 were significantly higher in severe than in moderate persistent asthma (*P* < .05). Remaining cytokines were similar in the subgroups.

### 3.5. Association between Duration of Asthma, Total Serum IgE, SPT, Cytokine Production, Lung Function Indices, Age of Patients, and Asthma Severity

**Figure 3:**
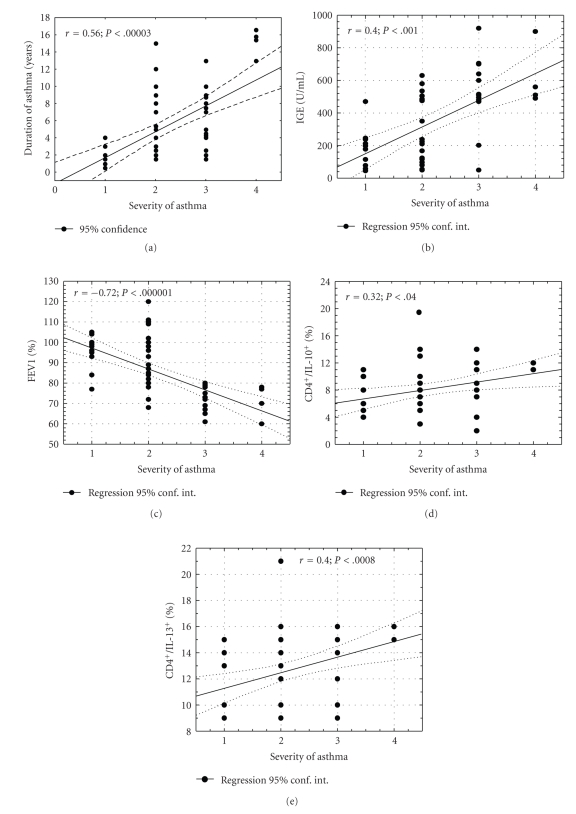
Scatterplot showing relationship between disease duration, total serum IgE, cytokine production, lung function indices, and the severity of asthma (degree of disease: 1-intermittent, 2-mild persistent, 3-moderate persistent, and 4-severe persistent asthma). Correlations were calculated using the Spearman test. (a) Duration of disease correlated highly significantly with severity of asthma (*r* = 0.56; *P* < .00003). (b) Positive correlation between total serum IgE and severity of asthma was revealed (*r* = 0.4; *P* < .001). (c) A highly negative correlation between severity of asthma and FEV_1_ was identified (*r* = −0.72; *P* < .000001). (d) There was a weak positive correlation between severity of asthma and the percentage of CD4^+^/IL10^+^

Severity of asthma was positively correlated with duration of asthma as well as serum IgE (*r* = 0.56; *P* < .00003 and *r* = 0.4; *P* < .001, resp.), but inversely with spirometric parameters such as FEV_1 _(forced expiratory volume in one second), FEV_1_/FVC, (FEV_1_ % forced vital capacity ), and MEF_25_ (maximal expiratory flow at 25% FVC) (*r* = −0.72; *P* < .000001, *r* = −0.56; *P* < .009, *r* = −0.68; *P* < .00001, resp.) (Figures 3(a), 3(b), and 3(c)). Moreover, severity of disease was positively correlated with the percentage of CD4^+^ T cells expressing IL-10 and IL-13 in response to PMA and ionomycin (*r* = 0.32, *P* < .04, *r* = 0.4, *P* < .008, resp.) (Figures 3(d), and 3(e)). No correlation was found between severity of asthma and age of children as well as degree of allergic sensitization expressed as total SPT wheal size (data not shown).

### 3.6. Relation between Cytokine Expression and Duration of Asthma

**Figure 4:**
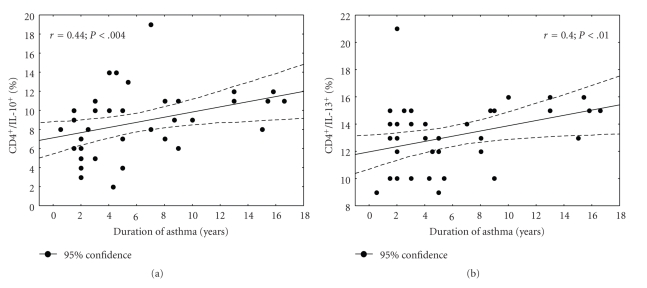
Scatterplot showing relationship between asthma duration and cytokine profile of peripheral blood T cells in asthmatic patients. (a) A good positive correlation was identified between percentages of CD4^+^/IL-10^+^ following stimulation and duration of asthma (*r* = 0.44, *P* < .004). (b) A good positive correlation was identified between percentages of CD4^+^/IL-13^+^ following stimulation and duration of asthma (*r* = 0.4, *P* < .01). Correlations were calculated using the Spearman test.

Frequencies of CD4^+^ T cells producing IL-10 as well as CD4^+^ T cells producing IL-13 was positively correlated with the duration of asthma (*r* = 0.44, *P* < .004 and *r* = 0.4, *P* < .01, resp.) (Figures 4(a), and 4(b)).

### 3.7. Relation between Dose and Duration of ICS Treatment and Cytokine Expression

No significant correlation between dose of ICS as well as duration of ICS treatment and cytokine expression in peripheral CD4^+^ and CD8^+^ T cells was found (data not shown).

## 4. Discussion

Using flow cytometric assay of the whole blood from children with asthma, we demonstrated significantly higher frequencies of both CD4^+^ and CD8^+^T cells expressing IL-4 and IL-13 compared with blood obtained from healthy, nonatopic children. Similarly to other reports, the excessive Th2 production by polyclonally stimulated T cells was accompanied by significantly decreased IFN-*γ* production in patients with asthma as compared to age-matched healthy controls [12, 13]. In this study, we also found higher frequencies of CD8^+^ T cells spontaneously producing IL-4 in children with asthma, which confirms previous findings from adult asthma patients, demonstrating increased levels of IL-4 in resting peripheral blood CD8^+^ T cells [14]. In the present study, we provided evidence that increased frequency of CD4^+^/IL-13^+^, but not CD4^+^/IL-4^+^ T cells was correlated with severity of asthma and these findings further suggest that IL-13 is a principal cytokine responsible for asthmatic symptoms. 

Increasingly, more evidence indicates that IL-13 plays a crucial role in the Th2 driven immunopathology and airway hyperresponsiveness seen in asthma [15–18]. IL-13 alone may mediate the main pathophysiological features of the disease, namely, mucus hypersecretion, subepithelial fibrosis, and bronchial hyperresponsiveness [15]. Moreover, some reports suggested that an increased IL-4 cytokine response is not necessarily a reflection of asthma activity but rather of the atopic state per se [19].

An increased expression of IL-13 in bronchial specimens, bronchoalveolar lavage fluid and cells, induced sputum, as well as in serum and allergen- or polyclonal-stimulated peripheral blood mononuclear cells from adult asthmatic patients have been reported by several authors [16–18, 20, 21]. Moreover, IL-13 levels in the airways correlated with disease severity in children and degree of airway hyperresponsiveness in adult asthma subjects, which may correspond well with our findings [16, 17]. 

Our study differs from the recent study by Antúnez et al. who reported that IL-4 and IL-13 production by circulating CD4^+^ and CD8^+^ T cells in response to phorbol 12-myristate 13-acetate and ionomycin was not significantly different in 15 children with mild asthma compared to healthy controls [9]. In the present study, a larger group of subject with different degree of disease was investigated, but no difference in IL-4 levels between intermittent, mild, moderate and severe persistent asthma was observed. In contrast, an increase in IL-13 in CD4^+^ T cells in severe asthma was revealed, though it was only significant as compared to intermittent asthma. An explanation for differences between our and Antunez study may be that asthma is heterogeneous disease. The recent finding suggests that asthma can be divided into at least two distinct molecular phenotypes defined by degree of Th2 inflammation, even in atopic subjects [22].

Similarly to other studies, a decrease in IFN-*γ* in our group of patients with asthma was confined to the CD4^+^ population of T cells, while CD8^+^ T cells did not show a significant decrease in IFN-*γ* [12]. It is believed that a defect in IFN-*γ* secretion following polyclonal stimulation is a general feature of atopic disease, and the role of IFN-*γ* in the pathogenesis of asthma is more complex. Indeed, in some studies, a significant decrease in IFN-*γ* in children and adults with severe asthma was found [23, 24] whereas a recent study in adult subjects suggest that the severity of asthma is associated with an increase in IFN-*γ* produced by CD8^+^ T cells [25].

In our study, we could not find any differences regarding IL-10 production by T cells between children with asthma and healthy controls, which is in line with the findings from other studies [9], but a trend towards higher cytokine level in the CD4^+^ T in children with severe asthma was observed. Unexpectedly, we also showed a weak correlation between IL-10 producing CD4^+^ T cells and the severity of asthma which may suggest that the expression of this cytokine is associated with aggravated allergic inflammation.

IL-10 is known to be secreted by many T cell subsets including CD8^+^ and CD4^+^ T cells. Unique populations of CD4^+^ T cells with regulatory properties; namely, CD4^+^CD25^+^FoxP3^+^ Treg cells and IL-10 producing Tregs have been identified [6]. Tregs distinct from conventional CD4^+^ T cells characterized by the expression of CD25 and intracellular FoxP3 protein were not assessed in the current study. On the other hand, IL-10 is a potent inhibitor of monocyte/macrophage function, suppressing the production of many proinflammatory cytokines and both Th1 and Th2 cell activation. There is increasing evidence that IL-10 secretion, in particular by Treg, may be defective in patients with asthma [26]. It was shown that T cells from children with asthma produce less IL-10 mRNA, and severe asthma in adult is associated with reduced frequency of IL-10 producing CD4^+^ T cells [27, 28]. In addition, lower concentrations of IL-10 in bronchoalveolar lavage (BAL) fluid or in induced sputum were found in subjects with asthma [29, 30]. On the contrary, other studies reported an increase in IL-10 levels in the serum or in the bronchoalveolar lavage fluid, [20, 31] as well as higher frequency of circulating IL-10—producing T cells in asthmatics compared with healthy subjects [32]. Moreover, paediatric patients with moderate to severe bronchial asthma had increased IL-10 mRNA expression in CD4^+^ T cells compared to those subjects with milder disease [33]. IL-10 may also promote airway hyperresponsiveness and eosinophilia in models of allergy [34]. 

 Our results also revealed that the severity of asthma was associated with increased total IgE levels, but not with the degree of sensitisation (i.e., skin prick test reactivity to a panel of allergens)_. _Moreover, a decrease in FEV_1,_ FEV_1_/FVC, and MEF-25 was observed in relation to the severity of asthma. Although most of the children in our study were regularly treated with ICS, we did not see a significant effect of dose or duration of ICS treatment on the profile of studied cytokines. On the other hand an increase in IL-10 and IL-13 in the CD4^+^, but not in the CD8^+^ T cells, was related with duration of asthma. In our study, although changes in cytokine profile secreted by CD8^+^ T cells in asthmatic subjects were found, they were independent of the severity of disease.

A possible limitation of our study is that it included children with a wide range of age, which although similar to that of the control group may bias the assessment of severity of asthma. Moreover, a cross-sectional design of our study limits the validity of our interpretation of the possible effect of ICS therapy on examined immunological parameters. Several studies have demonstrated that inhaled glucocorticoid treatment decrease Th2 cytokine and increase IL-10 mRNA and IFN-*γ* expression in peripheral blood T cells in moderate and severe asthmatic patients [10, 35]. The small numbers of the subjects with severe lung disease may also be a drawback of this study; nevertheless, some differences between severe asthma and other subgroups were found.

In conclusion, this study demonstrated dominant type 2-cytokine expressions in peripheral blood T cells in atopic asthma. Increased production of IL-4 and IL-13 in both CD4^+^ and CD8^+^ T cells accompanied by decreased IFN-*γ* expression in CD4^+^ T cells may be evidence that both lymphocyte subpopulations are implicated in the pathogenesis of asthma. In our study, although changes in cytokine profile in CD8^+^ T were found, they did not depend on the severity of the disease. Relationship of CD4^+^/IL-13^+^ T cells with disease activity suggests that this lymphocyte subset may have a prominent role in childhood asthma.

## Figures and Tables

**Table 1:** Demographic and clinical characteristics of patients with asthma and healthy children.

	Asthma	Healthy controls
*N*	40	18
Age, years, mean ± SE	9.02 ± 0.35	9.28 ± 0.75
Sex, *n *	M: 27; F: 13	M: 10; F: 8
Duration of asthma, years, mean ± SE	5.46 ± 0.54	
Serum total IgE, IU/ml, mean ± SE	312.95 ± 154.00*	28.20 ± 2.23
Eosinophils, % (*n*, cells/*μ*L), mean ± SE	7.2 ± 0.8 (260.4 ± 10.4)**	2.2 ± 0.2 (137.3 ± 6.2)
Positive skin prick test, *n* (%)	40 (100)	0
* Dermatophagoides pteronyssinus*	37 (92.5)	
* Dermatophagoides farinae*	38 (95)	
Grass pollens	21 (53)	
Tree pollens	12 (30)	
Weed pollens	15 (38)	
Moulds	10 (25)	
Feather	4 (10)	
Cat	8 (20)	
Dog	2 (5)	
Total SPT wheal size, mm, mean ± SE	21.9 ± 5.6	
Current inhaled steroid use, *n* (%)	30 (75)	
Lung function assessed, *n* (%)	35 (87.5%)	15 (83.3%)
FEV_1_% predicted	84.3 ± 2.06^#^	96.4 ± 2.047
FVC% predicted	83.3 ± 1.95^#^	91.71 ± 1.41
FEV_1_% FVC	87.47 ± 1.63^#^	91.97 ± 1.60
MEF_25_% predicted	85.85 ± 4.40^#^	95.85 ± 4.50

All *P*-values from Mann-Whitney U-test

*P < .01

**P < .001

^#^ *P* < .00001.

**Table 2:** Percentage of CD4^+^ and CD8^+^ T cells spontaneously producing IL-2, IL-4, IL-10, IL-13, IFN-*γ*, and TNF-*α*.

	CD4^+^/IL-2^+^	CD4^+^/IL-4^+^	CD4^+^/IL-10^+^	CD4^+^/ IL-13^+^	CD4^+^/IFN-*γ* ^+^	CD4^+^/TNF-*α* ^+^
Asthma *n* = 40	2.75 ± 0.17	3.45 ± 0.24	3.78 ± 0.26	3.78 ± 0.2	3.0 ± 0.18	2.92 ± 0.15
Controls *n* = 18	3.11 ± 0.31	2.83 ± 0.23	4.58 ± 0.71	3.33 ± 0.22	2.5 ± 0.26	3.0 ± 0.35

	CD8^+^/IL-2^+^	CD8^+^/IL-4^+^	CD8^+^/IL-10^+^	CD8^+^/IL-13^+^	CD8^+^/IFN-*γ* ^+^	CD8^+^/TNF-*α* ^+^

Asthma *n* = 40	3.4 ± 0.21	3.45 ± 0.19*	3.73 ± 0.31	4.4 ± 0.34	3.1 ± 0.19	3.53 ± 0.26
Controls *n* = 18	3.58 ± 0.36	2.75 ± 0.13	4.0 ± 0.48	3.42 ± 0.19	3.08 ± 0.4	3.0 ± 0.28

*P*-values from Mann-Whitney U-test

*P < .05

Data as shown as mean ± SE.

**Table 3:** Percentage of CD4^+^ T cells and CD8^+^ T cells producing IL-2, IL-4, IL-10, IL-13, IFN-*γ*, and TNF-*α* after in vitro activation with phorbol 12-myristate-13-acetate and ionomycin.

	CD4^+^/IL-2^+^	CD4^+^/IL-4^+^	CD4^+^/IL-10^+^	CD4^+^/IL-13^+^	CD4^+^/IFN-*γ* ^+^	CD4^+^/TNF-*α* ^+^
Asthma *n* = 40	7.78 ± 0.41	9.10 ± 0.34*	8.73 ± 0.55	13.1 ± 0.39*	7.83 ± 0.43**	7.73 ± 0.36
Controls *n* = 18	9.11 ± 0.56	6.17 ± 0.39	9.25 ± 0.69	7.83 ± 0.49	11.67 ± 0.58	7.22 ± 0.42

	CD8^+^/IL-2^+^	CD8^+^/IL-4^+^	CD8^+^/IL-10^+^	CD8^+^/IL-13^+^	CD8^+^/IFN-*γ* ^+^	CD8^+^/TNF-*α* ^+^

Asthma *n* = 40	8.08 ± 0.47	8.80 ± 0.28^$^	8.9 ± 0.5	13.00 ± 0.38*	7.65 ± 0.48	7.75 ± 0.49
Controls *n* = 18	8.08 ± 0.47	6.08 ± 0.40	8.67 ± 0.63	7.58 ± 0.53	8.50 ± 0.38	6.83 ± 0.44

*P*-values from Mann-Whitney U-test

*P < .00001,**P < .0001, ^$^ *P* < .00003

Data as shown as mean ± SE.

**Table 4:** Percentage of CD4^+^ T cells and CD8^+^ T cells producing IL-2, IL-4, IL-10, IL-13, INF-*γ*, and TNF-*α* before and after *in vitro* activation with phorbol 12-myristate-13-acetate and ionomycin in the four groups of asthmatics (1-intermittent, 2-mild persistent, 3-moderate persistent, and 4-severe persistent asthma). In parenthesis, the frequencies of spontaneous cytokine staining (in the presence of monensin alone) are presented.

	Subgroups of asthma patients			
		persistent asthma		
	1-intermittent *n*-10	2-mild *n*-14	3-moderate *n*-12	4-severe *n*-4
CD4^+^/IL-2^+^	7.5 ± 0.9 (3.2 ± 0.5)	7.5 ± 0.6 (2.8 ± 0.3)	9.1 ± 0.6 (2.3 ± 0.2)	6.9 ± 0.5 (3.0 ± 0.0)
CD4^+^/IL-4^+^	9.1 ± 1.1 (3.1 ± 0.3)	9.1 ± 0.4 (3.9 ± 0.5)	9.6 ± 0.4 (3.0 ± 0.4)	7.0 ± 0.41 (3.5 ± 0.3)
CD4^+^/IL-10^+^	7.1 ± 0.9 (5.0 ± 0.9)	8.9 ± 0.9 (3.5 ± 0.5)	8.5 ± 0.8 (3.0 ± 0.4)**	11.2 ± 0.2 (5.2 ± 0.2)
CD4^+^/IL-13^+^	11.5 ± 0.9 (4.0 ± 0.9)	13.0 ± 0.7 (3.5 ± 0.3)	13.3 ± 0.5 (3.4 ± 0.3)	15.2 ± 0.2* (5.2 ± 0.2)
CD4^+^/IFN-*γ* ^+^	6.8 ± 1.3 (3.3 ± 0.5)	7.9 ± 0.7 (3.0 ± 0.3)	7.0 ± 0.5 (2.7 ± 0.2)	7.2 ± 0.2 (3.0 ± 0.0)
CD4^+^/TNF-*α* ^+^	8.0 ± 0.9 (3.1 ± 0.3)	8.5 ± 0.5 (3.1 ± 0.2)	6.5 ± 0.6 (2.9 ± 0.1)	8.0 ± 0.4 (3.7 ± 0.0)
CD8^+^/IL-2^+^	7.5 ± 1.0 (3.5 ± 0.5)	8.5 ± 0.8 (3.5 ± 0.4)	7.3 ± 0.7 (3.1 ± 0.2)	9.2 ± 0.2 (4.7 ± 0.2)
CD8^+^/IL-4^+^	8.2 ± 0.9 (3.7 ± 0.5)	8.6 ± 0.4 (3.6 ± 0.3)	9.3 ± 0.4 (3.0 ± 0.2)	8.2 ± 0.2 (3.5 ± 0.2)
CD8^+^/IL-10^+^	7.5 ± 0.8 (3.8 ± 0.5)	9.7 ± 1.0 (4.3 ± 0.6)	8.2 ± 0.6 (3.3 ± 0.4)	10.0 ± 0.0 (2.2 ± 0.2)
CD8^+^/IL-13^+^	12.8 ± 0.9 (4.4 ± 0.6)	13.4 ± 0.7 (4.9 ± 0.7)	12.2 ± 0.5 (3.4 ± 0.3)	14.0 ± 0.5 (5.2 ± 0.7)
CD8^+^/IFN-*γ* ^+^	7.14 ± 1.0 (3.5 ± 0.9)	8.6 ± 0.91 (3.0 ± 0.3)	7.1 ± 0.6 (2.9 ± 0.3)	6.0 ± 0.0 (3.0 ± 0.0)
CD8^+^/TNF-*α* ^+^	7.57 ± 0.87 (3.7 ± 0.6)	9.1 ± 0.9^#^ (3.4 ± 0.4)	6.3 ± 0.6 (3.6 ± 0.4)	7.2 ± 0.2 (3.0 ± 0.0)

*P < .04 severe persistent asthma versus intermittent asthma.

**P < .05 severe persistent asthma versus moderate persistent asthma.

*P*-values from Mann-Whitney U-test.

Data as shown as mean ± SE.
